# Menthol cigarette smoking and nicotine dependence

**DOI:** 10.1186/1617-9625-9-S1-S5

**Published:** 2011-05-23

**Authors:** Allison C Hoffman, Dee Simmons

**Affiliations:** 1 Center for Tobacco Products, Food and Drug Administration, Rockville, MD 20850, USA; 2 Freelance medical writer, Fort Myers, FL, USA

## Abstract

Since tobacco use is driven by dependence on nicotine, the primary addictive substance in tobacco, much research has focused on nicotine dependence. Less well understood, however, is the role that menthol plays in nicotine dependence. This review seeks to examine what role, if any, menthol plays in nicotine addiction in adults and youth. Based on research examining several indicators of heaviness of nicotine addiction, including time to first cigarette upon waking, night waking to smoke, as well as some other indications of dependence, it is suggested that menthol cigarette smokers are more heavily dependent on nicotine. Although other indicators of nicotine dependence, including number of cigarettes per day and the Fagerstrom Test of Nicotine Dependence, failed to consistently differentiate menthol and non-menthol smokers, these indicators are thought to be less robust than time to first cigarette. Therefore, though limited, the existing literature suggests that menthol smokers may be more dependence on nicotine.

## Introduction

Tobacco use causes more than 440,000 deaths each year in the United States [1]. Nicotine is the primary addictive substance in tobacco, and nicotine dependence is a major factor that drives continued tobacco use. In fact, nicotine dependence promulgates tobacco use even after major health events such as hospitalization [2,3]. Menthol cigarettes make up a significant proportion of cigarettes sold in the United States, representing approximately one-fourth of all cigarettes sold [4]. This emphasizes the need to understand the potential impact of menthol on nicotine dependence; however, despite the significant literature on the chemical and pharmacologic properties of menthol [5,6], there is limited evidence surrounding the impact of menthol on nicotine dependence. This article reviews the available literature on menthol and nicotine dependence, and explored two questions:

• What is the effect, if any, of menthol on nicotine dependence in adults?

• What is the effect, if any, of menthol on nicotine dependence in youth?

Summarized in this review are 35 articles found to have either direct relevance to these questions, or were used to provide relevant background information. Many of these articles were identified through a review of the literature conducted by the National Cancer Institute in 2009, published as “Bibliography of literature on menthol and tobacco” (http://cancercontrol.cancer.gov/tcrb/documents/menthol_bibliography_508.pdf). Search terms used were menthol cigarette(s); mentholated cigarette(s); menthol tobacco; mentholated tobacco; menthol smoker(s); menthol AND the following terms: addiction, nicotine, marketing, cancer, biomarkers, asthma, cardiovascular disease, heart disease, vascular disease, chronic obstructive lung disease, respiratory, environmental tobacco smoke, national health, health disparities, and minority health. Additional searches and sources, such as those identified through review articles, identified additional articles that were included as appropriate. A publication or study is identified as having a tobacco industry association if one or more authors were employees of the tobacco industry, as identified by author affiliation on the publication.

Of those articles that are in the NCI Bibliography but were not included, most were not directly relevant to this topic (e.g., they studied menthol as a chemical independent from tobacco smoke exposure, did not evaluate menthol as a separate variable). Some of those articles, however, were used to provide background information. Animal or in vitro research was included only to help explain human findings. Although a few review articles were used to make general statements and/or provide background information, most were not included in deference to original sources. Published abstracts were not included out of concern that, due to the lack of details, those studies could not adequately be assessed.

## Menthol and nicotine dependence in adults

Time to first cigarette is a very robust, reliable indicator of strength of nicotine dependence. Those smokers who have their first cigarette of the day soon after waking up (e.g., ≤ 5 min) are considered to be more nicotine dependent than those who wait longer [7]. In a study of female smokers, smokers of menthol cigarettes had a shorter time to the first cigarette of the day as compared to non-menthol smokers (19.0 minutes and 37.4 minutes, respectively; p = .02) [8]. These data suggest that menthol cigarettes use may be associated with heavier nicotine dependence [7]. Another study by Ahijevych et al [9] compared female menthol smokers with non-menthol smokers, had similar results, but the results were not statistically significant (p = .085). A large retrospective cohort analysis of 1,699 patients of a cessation clinic found that 24.3% of menthol smokers had their first cigarette within 5 minutes upon waking as compared to 19.9% of non-menthol smokers (p = .021) [9]. Comparing Black/African American smokers with White smokers in an observational study, other researchers found that, on average, Black/African American smokers reported a shorter time to first cigarette after waking [10]. While menthol preference was discussed as a possible mitigating factor (78% of Black/African American subjects smoked menthol cigarettes vs. 5% of White subjects), the type of cigarette was not independently evaluated.

Not all studies have found a relationship between menthol smoking and shorter time to first cigarette. Hyland and colleagues (2002) found that one factor that was significantly associated with increased menthol use was greater than 60 minutes to the first cigarette in the morning; however, interpretation of this finding is difficult since smoking 4 or fewer cigarettes per day was also associated with increased menthol use [11].

Waking at night to smoke also appears to be a marker for tobacco dependence. Gandhi et al [13] conducted a retrospective cohort analysis of 1,688 consecutive patients who attempted to quit smoking. More menthol smokers than non-menthol smokers reported waking at night to smoke (55.3% and 44.9%, respectively; p < .001). Similar results were found by Bover et al [14], in a large study of more than 1,350 smokers at a tobacco dependence clinic. Menthol cigarette smokers (58%) reported waking at night to smoke compared with 45% of non-menthol cigarette smokers (p ≤ .0001). Furthermore, night-waking smokers had a significantly shorter time before smoking their first cigarette after waking in the morning, with 72% of menthol smokers reporting smoking their first cigarette of the day within five minutes or less, compared to 28% of non-menthol smokers (p ≤ .0001). Taken together, these data indicate that menthol smokers have greater nicotine dependence.

The number of cigarettes smoked per day (CPD) is also used as a measure of nicotine dependence. Whereas some research, including a tobacco-industry funded study, have reported that menthol cigarette smokers (particularly Black/African American individuals) smoke fewer CPD [13-15], others have failed to find significant differences [10-12]. Several studies that reported that Black/African American individuals smoked fewer CPD than did White smokers did not examine the independent effect of menthol cigarette use [8,12,19-25]. These conflicting results suggest no clear relationship between menthol cigarettes and cigarettes smoked per day; however, it should be noted that using CPD as a measure of nicotine dependence may have limitations since it can be significantly impacted by environmental constraints, such as no-smoking policies. It may also be influenced by the amount of nicotine in the cigarette, with fewer CPD needed to maintain nicotine levels. Some studies have shown that commonly used menthol cigarettes have significantly (p < .05) greater nicotine contents as compared to non-menthol cigarettes: 1.2 mg nicotine/cigarette and 1.0 mg nicotine/cigarette, respectively [26]. There may be a number of reasons why menthol smokers tend to smoke cigarettes with greater nicotine content, including differences in how menthol versus non-menthol cigarettes are designed or different blends of tobacco used in menthol versus non-menthol cigarettes. A tobacco industry associated study attempted to address the issue of possible design influences by creating experimental cigarettes using cased U.S. blended tobacco, normal manufacturing techniques, and identical design features. [27] The results indicated that the cigarette that included menthol flavoring had lower levels of nicotine per cigarette when compared to the unflavored, reference cigarette: 1.13 and 1.27 mg nicotine per cigarette, respectively [27]. Since the Baker et al [27] study indicates that menthol may somehow reduce nicotine content, the higher levels of nicotine seen in the previously discussed Okuyemi et al [26] study may be due to blend differences between menthol and non-menthol cigarettes which the menthol smokers find especially appealing.

The Fagerstrom Test for Nicotine Dependence (FTND) is an aggregate of several measures and is frequently used as a quantitative measure of nicotine dependence [28]. Included in this test are some of the most common measures of nicotine dependence, including measures of cigarette craving, as well as the previously discussed time to first cigarette and number of CPD smoked. Use of the FTND score may be limited because CPD accounts for 30% of the total FTND score and, as previously discussed, that may not be a very reliable measure of nicotine dependence. Using the FTND as an overall score for dependence, two studies failed to find an association between menthol cigarette use and non-menthol cigarette use [26,29]. In other studies, researchers found that Black/African American smokers score higher on the FTND, [28,30] however, menthol cigarette use was not evaluated as a separate factor.

In summary, there were four studies that investigated the effect of menthol on time to first cigarette. One study found that menthol smokers had significantly shorter times as compared to non-menthol smokers [8], one found a trend towards menthol smokers having a shorter time to first cigarette [9], and one found that greater than 60 minutes to first cigarette was associated with menthol use (possible confound of menthol use being associated with ≤ 4 CPD) [11]. Additionally, night waking menthol smokers had a significantly shorter time before smoking their first cigarette after waking in the morning [14]. There were two studies that found that menthol smokers were more likely to wake at night to smoke [13,14]. There were mixed findings on CPD, with three studies finding that menthol smokers smoked fewer CPD [13-15], and three finding no significant differences [10-12]. Two studies failed to find any differences in FTND scores [26,29]. In general, if differences were seen, they were in the direction of indicating that menthol smokers show greater signs of nicotine dependence.

## Menthol and nicotine dependence in youth

Smoking typically begins in youth, with regular use usually starting between the ages of 14 and 21 [31]. Data from two national, school-based surveys of adolescents in grades 6 through 12 (the National Youth Tobacco Survey [NYTS]) were used to assess the relationship between menthol cigarette use and nicotine dependence in youth [32]. After controlling for demographic background and the length, frequency, and level of smoking, logistic regression showed that teens who regularly smoked menthol cigarettes had 45% greater odds of scoring higher on a nicotine dependence scale than teens who regularly smoked non-menthol cigarettes (OR = 1.45, p = .006). The authors suggested that smokers may find it easier to inhale more deeply from menthol cigarettes and therefore take in more nicotine [32].

Wackowski and Delnevo [33] also analyzed data from the 2004 NYTS and examined measures of nicotine dependence among adolescent smokers of menthol and non-menthol cigarettes were examined. The focus was on high school students who were already established smokers. After controlling for demographic factors and smoking patterns, compared with smokers of non-menthol cigarettes, smokers of menthol cigarettes were more likely to report symptoms of dependence, such as needing a cigarette less than one hour after smoking (OR = 2.6; CI = 1.6–4.3) and experiencing craving after not smoking for a few hours (OR = 1.6; CI = 1.1–2.2). The researchers concluded that young smokers of menthol cigarettes may experience greater withdrawal and addiction symptoms as compared to young smokers who smoke non-menthol cigarettes. There were several limitations to the study: data were based on self-reports, which may result in underreporting or overreporting, and the design of the survey allowed for contradictory responses.

Adolescent smokers, most of whom smoked menthol cigarettes (531 out of 572 smokers), were asked how soon after waking up they smoked their first cigarette. The smokers of menthol cigarettes in this cross-sectional study were more likely (45%) than smokers of non menthol cigarettes (29%) to smoke their first cigarette within 5 minutes of awakening [10]. However, menthol cigarette use did not significantly affect the number of cigarettes smoked per day or scores on the FTND. The findings related to menthol cigarette use and time to first cigarette upon waking for the day, CPD, and FTND parallel those found in adults [8,26,29]. Although the limitations of CPD and FTND were previously discussed, additional caution may be warranted when interpreting these findings in youth smokers. Adolescents are subject to greater social restrictions on smoking than adults, including school and household rules and restricted access to obtaining a cigarette.

DiFranza et al [34] posed a sensitivity model for predicting the development of nicotine dependence in a retrospective study on the recollections and repercussions of the first ever inhaled cigarette in a cohort of 237 students from two urban school systems. The strength of subsequent addiction did not differ according to the brand, brand strength, or menthol content of the first cigarette. Limitations of the study included the subjective nature of the symptoms studied, limited age range of the subjects, natural variability in nicotine dosing, and the reliance upon youths’ memories regarding details of their first smoking experience.

In summary, results from three studies indicate that youth menthol smokers may be more dependent as compared to youth non-menthol smokers, including scores on a nicotine dependence scale [32], likelihood of reporting symptoms of dependence [33], and shorter time to first cigarette [33]. Two studies failed to find significant differences in CPD, FTND or other addiction measures [10,34]. As with studies of adult menthol smokers, if differences were seen, they were in the direction of indicating that youth menthol smokers show greater signs of nicotine dependence.

## Nicotine exposure and nicotine dependence

Nicotine metabolism is important to consider as an underlying factor that may influence nicotine dependence. When smoke is inhaled from a cigarette, nicotine contained in the tobacco smoke passes into the lungs and is rapidly absorbed into the circulatory system and delivered to the brain within seconds. Nicotine metabolized into cotinine, which has a longer half-life than nicotine and is therefore often used as a proxy measurement of nicotine exposure. People who metabolize nicotine more slowly have reduced nicotine clearance, leading to more sustained exposure of nicotine [35].

It has been postulated that smokers of menthol cigarettes may not need to smoke as many CPD to maintain the desired level of nicotine in the body; however, the data regarding menthol’s effects on nicotine metabolism are inconclusive. There is an alternative possibility for why menthol smokers have been found, in some cases, to smoke fewer CPD. In several studies, menthol smokers have been found to have higher cotinine/cigarette ratios as compared to non-menthol smokers [8,9,18], indicating that they are taking in more nicotine per cigarette. This increase in nicotine exposure may be due to differences in smoking behavior or that the cigarettes that menthol smokers are smoking may contain higher levels of nicotine per cigarette, as compared to those smoked by non-menthol smokers: 1.2 mg/cigarette compared to 1.0 mg/cigarette [26].

## Summary

The majority of indicators of nicotine dependence, including time to first cigarette upon waking (youth and adults), night waking to smoke (adults), and some other indications of dependence (youth) suggest that menthol cigarette smokers are more heavily dependent on nicotine. Although some other indicators of nicotine dependence, including CPD and FTND, failed to consistently differentiate menthol and non-menthol smokers, these indicators are not thought to be as robust as time to first cigarette.

## Competing interests

The authors declare that they have no competing interests.
